# Diode laser surgery for the treatment of denture-induced fibrous hyperplasia: a case report

**DOI:** 10.11604/pamj.2024.47.105.26619

**Published:** 2024-03-05

**Authors:** Er-Raji Samir, Rokhssi Hasnae, Ennibi Oumkeltoum

**Affiliations:** 1 Department of Periodontology, Faculty of Dentistry of Rabat, University Mohamed V, Rabat, Morocco

**Keywords:** Hyperplasia, diode laser, surgery, case report

## Abstract

Denture-induced fibrous hyperplasia (DIFH) is a persistent lesion caused by low-intensity chronic injury of the tissue in contact with an ill-fitting, over-extended denture. This fibrous connective tissue lesion commonly occurs in oral mucosa in patients showing important alveolar ridge atrophy. Surgical excision is the treatment of choice for DIFH. This article describes a successful laser surgery to remove a DIFH on a lower alveolar ridge of a patient wearing an ill-fitting completely removable denture. The use of a diode laser may result in less surgical time, less bleeding during surgery, more vestibular depth, better re-epithelialization of the wound, and no need for suturing.

## Introduction

Denture-induced fibrous hyperplasia (DIFH), also known as epulis fissuratum, typically manifests around the borders or flanges of complete or partial removable dentures, whether in the maxilla or mandible. This condition is often characterized as a raised, sessile, or pedunculated lesion consisting of dense fibrous connective tissue. It develops as a result of chronic trauma and inflammation induced by the pressure exerted by the denture flange. Studies have indicated that the majority of cases occur in individuals aged between 50 and 60 years (37.7%), with a higher prevalence in women (81.2%) and an equal occurrence rate observed between the maxilla and mandible [1].

The treatment of this kind of lesion includes elimination of etiologic factors and surgical removal of the lesion. The most common techniques used for removing this hyperplastic lesion are surgical scalpels, electrical scalpels, or different types of lasers [2]. In the last decades, the use of laser has resulted in many significant improvements compared to conventional surgical methods: minimal bleeding, greater precision, better site decontamination, less oedema, reduced postoperative pain, and no need for sutures [3,4]. The most commonly used laser in oral surgery is the diode laser due to its small size, low cost, and ease of use for minor soft tissue surgery.

## Patient and observation

**Patient information:** a fifty-three-year-old woman presented at the Department of Periodontics of Dental Clinic at Mohammed V University, Rabat, Morocco, with the chief complaint of an abnormal growth along the anterior border of her ill-fitting mandibular complete denture.

**Figure 1:**
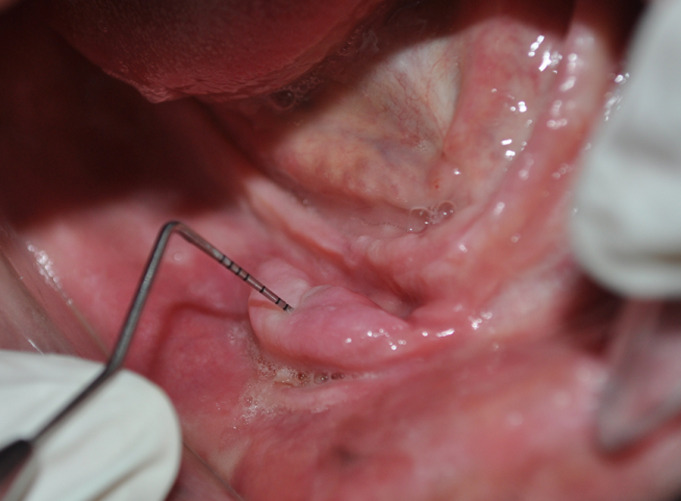
denture-Induced fibrous hyperplasia over the alveolar ridge extending to the vestibular sulcus in the lower anterolateral region

**Figure 2:**
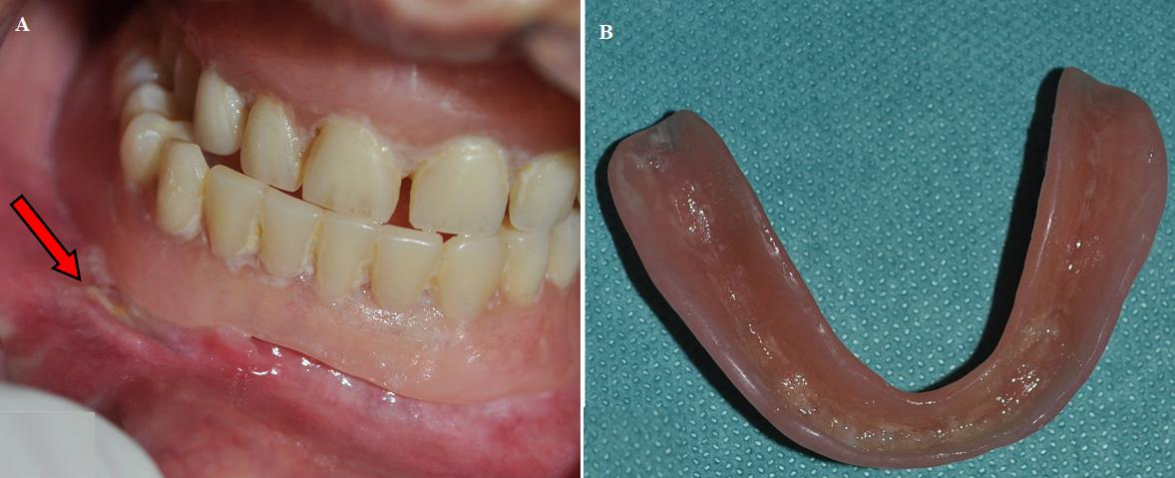
A) ulceration in contact with the edge of ill-fitting prosthesis; B) complete lower denture with very fine edges

**Clinical findings:** clinical examination showed a completely edentulous ridge with a hyperplastic lesion extending to the vestibular sulcus in the lower anterolateral region, sized about 1 cm × 2.5 cm (Figure 1). Also, an ulceration is observed in contact with the edge of an old ill-fitting prosthesis (Figure 2). No relevant medical history, and did not use any medications.

**Diagnosis:** the list of potential differential diagnoses for the lesion included irritation fibroma, peripheral giant cell granuloma, peripheral ossifying fibroma, bony exostosis, pyogenic granuloma, benign mesenchymal tumors, and minor salivary gland tumors. Based on the patient's medical history and clinical examination findings, a provisional diagnosis of denture-induced hyperplasia was established, and an excision biopsy using a diode laser was planned.

**Figure 3:**
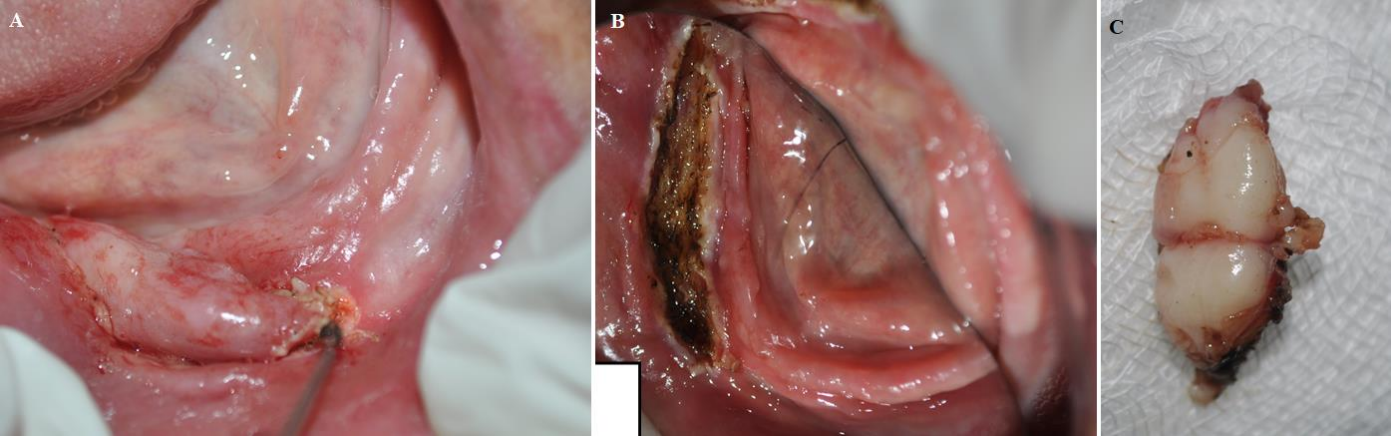
A) intraoperative: excision with diode laser; B) immediate postoperative view after laser excision; C) clinical view of the denture-induced fibrous hyperplasia after laser excision

**Figure 4:**
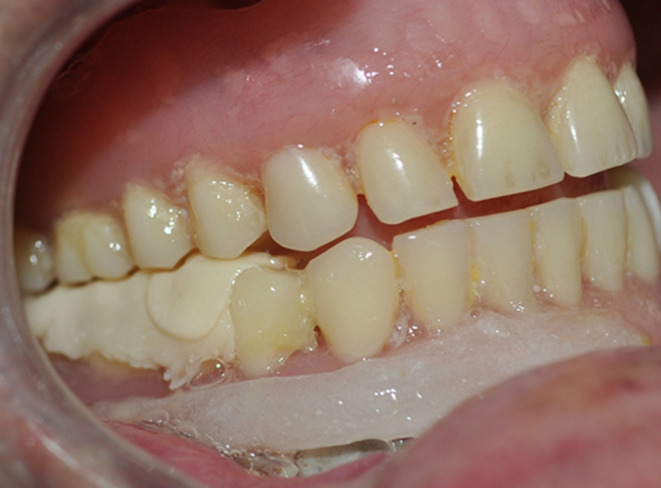
the lower denture was rebased with a soft tissue conditioner

**Therapeutic interventions:** the area surrounding the lesion was infiltrated with local anesthetic (lignocaine 2% with adrenaline 1: 80,000). The lesion removal was made using a diode laser (doctor smile dental laser 980 nm by LAMBDA SpA Italy) using a fiber of 300-μm in contact mode and pulse of 30 ms duration and 30 ms interval with initiated tip at 3 W power (Figure 3). Dentures were repositioned, with the immediate lining of the mandibular one using a soft tissue conditioner to stabilize it and facilitate wound healing (Figure 4). The excised lesion was put in 10% formalin and sent for histopathological examination, which showed hyperplastic epithelium as well as a fibrous connective tissue with moderate inflammation.

**Figure 5:**
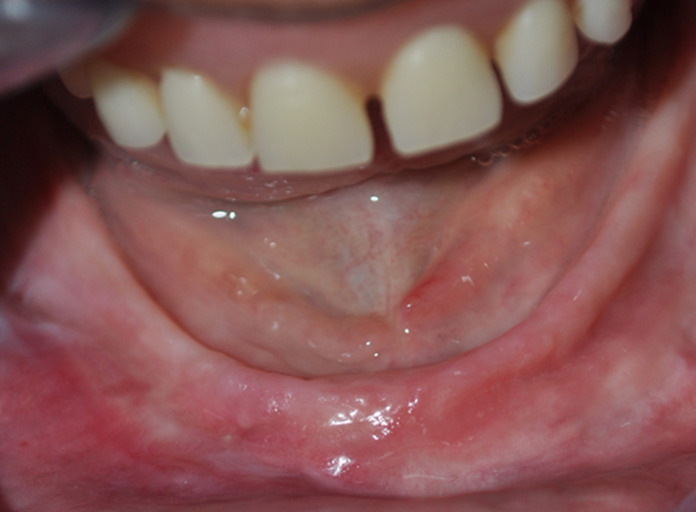
the aspect of the post-surgical area after 2 months

**Follow-up and outcome of interventions:** after 1 week, satisfactory tissue took place. After 2 months, complete resolution without any recurrence was observed (Figure 5). Finally, the patient was referred to the department of prosthodontics for proper prosthetic rehabilitation.

**Patient perspective:** after excision of the gingival hyperplasia, the patient experienced a little discomfort in the following hours, for which the patient did not find it necessary to take painkillers. However, he was relieved that the procedure went well, especially that he had never heard of laser before. Upon finding a stable prosthesis immediately after surgery, he became determined to follow his dentist's instructions to ensure a complete recovery.

**Informed consent:** the patient was informed in detail about the procedure after which he signed an informed consent form.

## Discussion

Fibrous hyperplasia induced by dentures (DIFH) is a condition characterized by excessive growth of fibrous connective tissue in the oral mucosa, typically resulting from trauma. This trauma often occurs due to poorly fitting denture borders, leading to chronic, low-intensity injury [5]. Other contributing factors may include parafunctional habits, inadequate oral hygiene, and smoking. In the case we have documented, the presence of an ill-fitted denture and poor oral hygiene were identified as contributing factors. Clinically, DIFH manifests as painless, raised, smooth, pinkish folds of hyperplastic mucosa, typically found in the gingivolabial or gingivobuccal sulci, particularly in the anterior region of the mouth. The size of these lesions can vary depending on the severity of the trauma. In cases of more intense trauma, the surface of the lesions may become ulcerated, leading to significant discomfort for patients [6].

Treatment options for DIFH include both conservative and surgical approaches. In the initial stages of fibrous hyperplasia, characterized by minimal fibrosis, conservative treatment such as adjusting the fit of the denture is often effective in reducing or eliminating the excess tissue. However, in cases where significant fibrosis has developed, surgical removal of the hyperplastic tissue is the preferred treatment. Following surgical excision, it is essential to create a new denture for the patient and educate them on maintaining proper oral hygiene practices [7].

When surgical intervention is necessary for these lesions, various procedures such as conventional scalpels, electric scalpels, or different types of lasers can be employed. In a study by Amaral *et al*. a comparison was made between diode laser and scalpel surgery for the treatment of fibrous hyperplasia [4]. The study concluded that diode laser surgery offers several advantages over conventional scalpel surgery, including superior incision performance, effective coagulation, reduced postoperative pain and swelling, and minimized scarring. Additionally, lasers provide instant disinfection of the surgical wound. Furthermore, Amaroli *et al*. have suggested that utilizing high-power diode lasers can deliver sufficient light doses to penetrate deeper tissues and induce photobiomodulation. These heightened metabolic activities accelerate cellular proliferation and migration, thereby promoting faster wound healing [8].

The hyperplastic tissue typically arises from an inflammatory process; nevertheless, it's crucial to recognize that other pathological conditions may be present. Therefore, it is imperative that representative tissue samples are always submitted for pathological examination following removal.

Monteiro *et al*. reported that lasers can effectively excise oral benign fibrous-epithelial hyperplasias, with no limitations regarding histopathological diagnosis, provided that the physical properties of each laser are understood and adhered to. They also found that the instrument causing the most extensive tissue damage was the electrosurgical scalpel, followed by the diode laser, Nd: YAG laser, CO2 laser, and lastly, the Er: YAG laser [9]. Angiero *et al*. concluded that the diode laser can induce significant thermal effects in small lesions. They recommended that specimens should be at least 5 mm in diameter to ensure a reliable evaluation of the histological sample [10].

## Conclusion

Diode lasers can be an excellent alternative to conventional therapeutics. This case demonstrates good hemostasis at surgical area with no need for suture, an absence of infection, and an effective post-operative comfort.
